# A ‘fishy’ ECG in a patient with chest pain

**DOI:** 10.1007/s12471-021-01632-0

**Published:** 2021-09-15

**Authors:** V. Devesa Neto, J. M. Santos, J. G. Pereira, L. Ferreira Santos, B. Marmelo

**Affiliations:** Cardiology Department, Tondela-Viseu Hospital Centre, Viseu, Portugal

## Answer

**Fig. 1:**
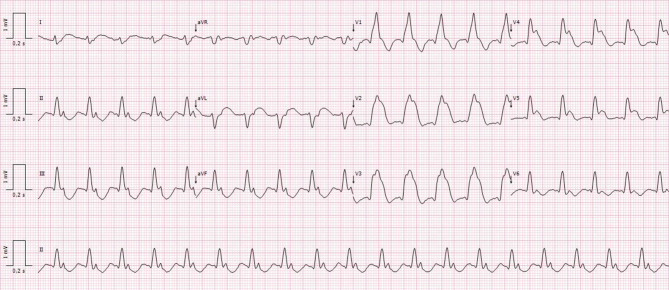
Electrocardiogram at hospital admission

**Fig. 2:**
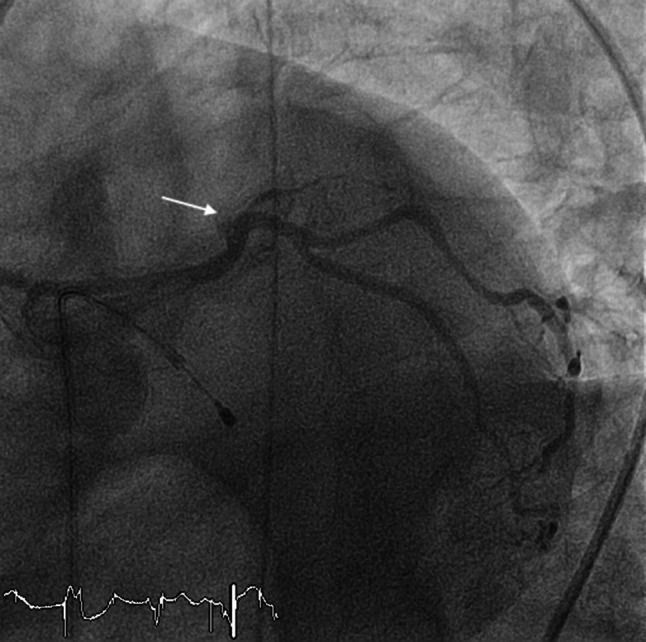
Emergent coronary angiography revealing occlusion of ostial left anterior descending artery (*arrow*) seen in left caudal view

The triangular QRS-ST‑T waveform (TW) pattern, also called ‘tombstone’ or ‘graveyard’ and more recently ‘shark fin’ pattern, is a rare electrocardiography pattern that is associated with a poor prognosis in patients with ST-segment elevation myocardial infarction [1–3]. The ECG (Fig. 1 in the question) showed supraventricular tachycardia and an anterior TW pattern. Echocardiography at hospital admission revealed changes in segmentary motility of the left ventricle corresponding with descending artery territory, and the left ventricular systolic function was severely reduced. No evidence of mechanical complications was found. The patient was referred to urgent coronary angiography, which revealed occlusion of the ostial left anterior descending artery (Fig. 2). After multiple unsuccessful attempts to cross the thrombotic lesion with the guidewire, the patient had another cardiopulmonary arrest. ‘Life-saving’ intracoronary fibrinolysis with alteplase during resuscitation manoeuvres was attempted, without success.

A TW pattern is an unusual finding as these patients usually die rapidly, before being able to get medical assistance and before an ECG is recorded. It is of the utmost importance that medical personnel recognise this pattern immediately, because this allows for an urgent response, including early reperfusion.
